# Analysis of DICOM Image Compression Alternative Using Huffman Coding

**DOI:** 10.1155/2019/5810540

**Published:** 2019-06-17

**Authors:** Romi Fadillah Rahmat, T. S. M. Andreas, Fahmi Fahmi, Muhammad Fermi Pasha, Mohammed Yahya Alzahrani, Rahmat Budiarto

**Affiliations:** ^1^Department of Information Technology, Faculty of Computer Science and Information Technology, Universitas Sumatera Utara, Medan 20155, Indonesia; ^2^Department of Electrical Engineering, Faculty of Engineering, Universitas Sumatera Utara, Medan 20155, Indonesia; ^3^Malaysia School of Information Technology, Monash University, Bandar Sunway 47500, Malaysia; ^4^College of Computer Science and Information Technology, Albaha University, Al Bahah, Saudi Arabia

## Abstract

Compression, in general, aims to reduce file size, with or without decreasing data quality of the original file. Digital Imaging and Communication in Medicine (DICOM) is a medical imaging file standard used to store multiple information such as patient data, imaging procedures, and the image itself. With the rising usage of medical imaging in clinical diagnosis, there is a need for a fast and secure method to share large number of medical images between healthcare practitioners, and compression has always been an option. This work analyses the Huffman coding compression method, one of the lossless compression techniques, as an alternative method to compress a DICOM file in open PACS settings. The idea of the Huffman coding compression method is to provide codeword with less number of bits for the symbol that has a higher value of byte frequency distribution. Experiments using different type of DICOM images are conducted, and the analysis on the performances in terms of compression ratio and compression/decompression time, as well as security, is provided. The experimental results showed that the Huffman coding technique has the capability to compress the DICOM file up to 1 : 3.7010 ratio and up to 72.98% space savings.

## 1. Introduction

DICOM (Digital Imaging and Communication in Medicine) is a file standard used to handle, store, print, and send information in medical imaging. All modern medical imaging devices (imaging modalities) such as X-ray, CT (computed tomography) scan, and MRI (magnetic resonance imaging) use DICOM as their standardized file output. A DICOM file consists of a few data elements or attributes capable to store some information, such as patient data (name, sex, etc.), imaging procedure (calibration, radiation dose, contrast media, etc.), and the information of the image itself (resolution, pixel data, bit allocation, etc.) [1]. Due to its bigger size than the other standard sizes of the image file, the storage and transmission of the DICOM file become one of the problems in an integrated hospital information system (HIS) with picture archiving and communication system (PACS) implementation. The larger the size of the data, the more the storage media and bandwidth for the data transmission are required. It certainly causes the problem in terms of procurement cost for larger storage and bandwidth [2–4].

Data compression is one of the solutions to overcome this problem. Data compression is to convert the input data source into the output data that has a smaller size [5]. The main purpose of compression techniques is memory efficiency, fast compression, generation of the best output. It can be divided into two types, namely, lossless compression and lossy compression. Lossless compression is a type of data compression which does not remove any information from the initial data, while the lossy compression removes some of the information from the initial data [6].

Lossy data compression is usually used for generating higher compression ratio, without considering the loss of information in the image [7]. The latest research on lossy data compression was conducted by Kumar et al. [8] who used a logarithm method called LDCL (lossy data compression logarithm) in their methodology. Their experimental results showed that the particular method could generate the compression ratio up to 1 : 60 in many cases.

The lossless JPEG2000 is the popular data compression method used in various PACS and considered the standard for DICOM compression [9] despite being not backward compatible [10]. Nevertheless, ongoing researches are still being carried out to analyze the performance of JPEG2000 compression method as well as proposing an alternative compression method in PACS with the aim to balance image quality and transfer duration [9, 11–15]. Thus, this work implements and provides the performance analysis of the Huffman coding, identified as one of the lossless standard data compression methods by the US Food and Drug Administration (FDA) [16].

Existing work on Huffman coding adoption to compress the DICOM image by Kavinder [17] did not address the performance, security aspect, complexity, and compression time for compressing the DICOM image file by considering the information stored in the file.

## 2. Related Work

Huffman coding has been used for many cases of data compression. In 2015, Ezhilarasu et al. [18] reviewed Huffman coding and concluded that the Huffman code can provide better compression ratio, space savings, and average bits than uncompressed data. A comparative study was performed by Maan [19] in 2013, who analyzed and compared three lossless data compression codings, namely, Huffman, arithmetic, and run length. The experimental results showed that arithmetic coding can generate highest compression ratio among lossless data compression techniques, but its compression speed is slower than the Huffman coding.

Another related research work was done by Medeiros et al. in 2014 [20]. They compressed lightweight data for wireless sensor networks (WSNs) by monitoring environmental parameters by using low-resolution sensors. The obtained percentage of the compression ratio in their experiment varied from 46% to 82%. The researchers stated that the Huffman coding is extremely simple and outperforms lossless entropy compression (LEC) and adaptive linear filtering compression (ALFC) in most cases.

Research has been conducted on DICOM image file compression using various techniques. In fact, several studies combined lossless and lossy data compression techniques. In 2013, Kavinder [17] combined Huffman coding (lossless) and discrete cosine transform (lossy) and improved the technique by using vector quantization to increase the compression ratio. In 2015, Kumar and Kumar [21] used hybrid techniques of discrete wavelet transform-discrete cosine transform (DWT-DCT) and Huffman coding, while Fahmi et al. introduced sequential storage of difference for image compressing in medical image cloud application [22, 23]. Other works on lossless and lossy data compression techniques are found in [24–28].

## 3. Materials and Methods

In previous studies, the lossy data compression technique generates high compression ratio but decreases the quality metrics of the peak signal-to-noise ratio (PSNR), which is generally used to analyse the quality of an image. The higher the PSNR is, the better the quality of the compressed or reconstructed image is. Thus, the lossless technique should be applied for the enhancement of the compression of the same PSNR. An image can be compressed without the loss of significant details through Huffman coding. In the perspective of a DICOM file, we expect that the DICOM image has intact quality and metadata after its compression and decompression. Standard DICOM compression method is in JPEG2000, and thus, we compare the performance analysis between JPEG2000 and Huffman coding as an alternative DICOM compression method. The lossless criteria of Huffman coding are the foundation of this work. Image quality after decompression is a vital point here and is the reason for selecting Huffman coding as the methodology.

**Figure 1:**
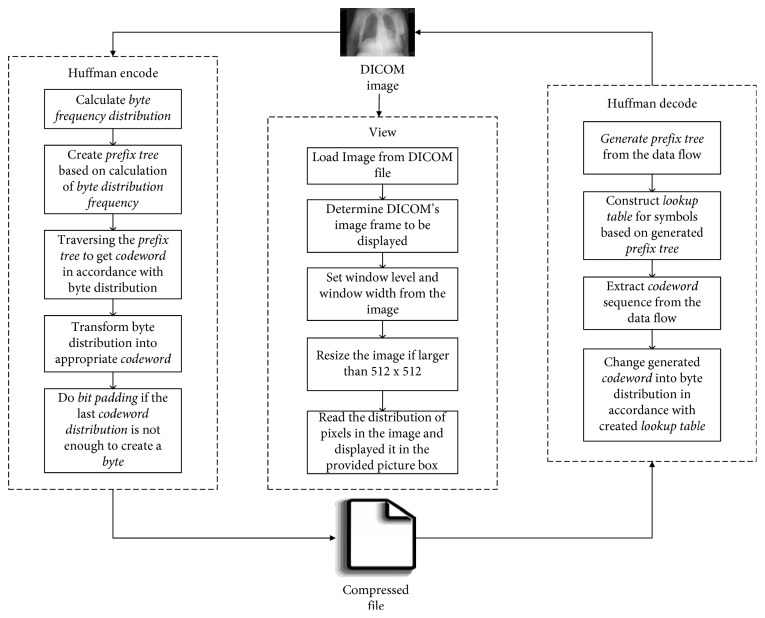
General architecture.

Figure 1 shows the three parts of the methodology. The first part is Huffman encoding for DICOM image file compression. The DICOM image file is collected first and used as a source. This part encodes (compresses) the file by calculating the byte frequency distribution (BFD), creating a prefix tree to get codewords, changing byte distribution into codewords, and then performing bit padding if necessary. The second part is View, which displays the DICOM image and includes the steps for determining the image frame and window level and width and for resizing the image. The third part is Huffman decoding for decompression. In the decoding process, a prefix tree is read from a compressed data flow file, and codeword threads are extracted from data flow and changed back into the original distribution byte. The more detailed steps in the methodology are described below.

### 3.1. Huffman Encode

The input DICOM file is compressed in the first step. The byte distribution on file is read and calculated with BFD. Next, a prefix tree is created for the acquisition of codewords that will substitute the byte distribution on the input file used for generating a new smaller file. The data used for encoding are the byte distributions, which are compiled in the DICOM file, as shown in the following (in hexadecimal):

  00 00 00 00 00 00 00 00 00 00 00 00 00 00 00 00
  44 49 43 4d 02 00 00 00 55 4c 04 00 be 00 00 00
  82 04 82 04 82 04 82 04 82 04 82 04 82 04 82 04
  7d 04 78 04 78 04 78 04 78 04 78 04 69 04 5a 04
  5a 04 00 00 00 00 00 00 00 00 00 00 00 00 00 00

The BFD, which is a table storing the frequency of occurrence value from every byte that compiles the file, is calculated for each DICOM file. For example, when the FF byte (255) occurs 887 times in a file, the value of the FF byte (255) in the BFD table is 288700.  Table 1 shows the BFD from one of the DICOM files used in the experiment. If the FF byte (255) occurs 200 times in a file, then the value of the FF byte (255) in the BFD table is 200.

Once the calculation of BFD is completed, a prefix tree is created for the acquisition of appropriate codewords as substitutes of byte distribution [6].  Table 2 shows the result of the generated codewords after searching the prefix tree, which was created on the basis of BFD in Table 1.

Bit padding is the process of adding one or more bits into the data flow to fit into the minimum 8-bit computer architecture. In the example, when the generated data size is 7 bit, then 1-bit padding is required to fulfill the 8 bit (1 bytes), and if the generated data size was 28 bit, then 4-bit padding is required to fulfill the 32 bit (4 byte), and so on.

### 3.2. View

The View part comprises the features that are built as the user interface. This part shows to the user the compressed DICOM image, which is displayed in accordance with the original size or the size of the provided canvas. This part is called the DICOM Viewer in other systems. The View loads the image from the DICOM file, determines the DICOM image frame to be displayed and the window level and width to be configured, resizes the image, and reads and displays the pixel distribution of the image. The View describes the metadata of the DICOM file.

### 3.3. Huffman Decode

The Huffman decode decompresses the file to be restored in its original file. The part starts with the reading of the data flow of the compressed file, creation of a prefix tree from previously stored data into data flow in the encoding process, and construction of a lookup table that contains codewords and symbols to be changed back into the original byte structure before the compression [29]. However, using the bottom-up Huffman tree with probabilities [30] is good in terms of run time; however, for the case of DICOM compression, we found that using a lookup table provide a balance between faster run time and memory usage.

The lookup table contains codewords and represents symbols generated from the results of the search of the prefix tree created previously. The table is used for changing the codeword thread from the data flow back into the original byte distribution.  Table 3 shows the lookup table for the compressed DICOM file, CT0011.dcm (CT0011.huf).

After reading the codeword thread of the compressed file data flow, the generated codeword is changed into an original byte distribution or symbol according to the created lookup table. This step is conducted by reading every bit of data flow, then determining whether the bit is in the lookup table. If not, the next bit is read and threaded with the previous bit; then, the search is repeated in the lookup table. If the thread of bit is found as one of the codewords that represents a symbol, then the bit is changed into the symbol. The process is done continuously until the number of the returned symbols achieve the original data size.

The programming language used in this research is VB.NET, while the operating system used is Windows 7 Ultimate 64 bit SP1. The computer specification is Intel i5 450M in processor unit, 4 GB RAM, 500 GB HDD, and ATI Radeon HD 5470 in graphic card.

The data used in this research work are several sets of DICOM image files available at http://www.mmnt.net/, and another set of the anonymized DICOM image from computed tomography is collected randomly from the University Hospital RS USU Medan. The files used were 20 DICOM image files with the extension ∗.dcm. The specifications of all DICOM files are described in Table 4.

## 4. Results and Discussion

### 4.1. Huffman Coding Compression Performances

Tables 5 and 6 present the results of DICOM file compression through the Huffman coding technique. The specifications are provided in Table 4. From the obtained result, the percentage of space savings is up to 72.98% at a 1 : 3.7010 compression ratio, while the lowest space saving percentage is at −0.08%. The worst compression ratio is 1 : 0.9992.

One of the factors that affect the compression ratio is the number of nodes or symbols, which creates a prefix tree of the image file. The tree is shown in CT-MONO2-16-ankle.dcm and CT-MONO2-16-brain.dcm files, which nearly have the same original size (±525 kB) but have different compression ratios. The CT-MONO2-16-ankle.dcm file was twice as large as the CT-MONO2-16-brain.dcm file with respect to the compression ratio.

Compared to other image data sets in Table 6, the CT0013.dcm file has a smaller compression ratio than that of the CT0014.dcm file, although the former had fewer symbols. Hence, another factor affects the value of the compression ratio apart from the number of symbols. One such factor is BFD value of the file. The BFD values of the CT0013.dcm and CT0014.dcm file are shown in Table 7.

The BFD of the CT0013.dcm file spreads more evenly than that of the CT0014.dcm file. This feature causes the length of the codeword from the former to exceed that of the latter. For instance, if the assumption for the length of codewords for byte 00 is 2, byte 03 is 5, and byte FE is 6, then the obtained size of CT0013.dcm file when other bytes are disregarded is as follows:

(1) $$\begin{array}{c} \left(663477\times 2+24806\times 5+24489\times 6\right)=1597918\text{ bit}\approx 199740\text{ byte}, \end{array}$$

while the obtained size of CT0014.dcm file is as follows:

(2) $$\begin{array}{c} \left(698830\times 2+14\times 5+91\times 6\right)=1398276\text{ bit}\approx 174785\text{ byte}. \end{array}$$

In the experimental results, we also observe a limitation of Huffman coding where one case generates a compression ratio of less than 1 (ratio < 1), causing the generated compressed file size is larger than the original file size.

Table 8 shows the BFD pattern and the codeword from CT-MONO2-16-chest.dcm file, which has 1 : 0.992 of compression ratio. The BFD value is nearly evenly distributed and thus causes the created prefix tree to generate codewords, which are approximately equal to or longer than the required bit length for the creation of 1 byte (8 bit). For the CT-MONO2-16-chest.dcm file, the created codewords are 11 (7 bit long), 19 (9 bit long), and 2 (10 bit long). The other codewords are 8 bit long. From the total number of created codewords, only 11 symbols are compressed into 7 bits, while the others still have the 8 bit length or more (9 bit and 10 bit). Hence, the compression of CT-MONO2-16-chest.dcm file generates a larger file size than the original size.

**Figure 2:**
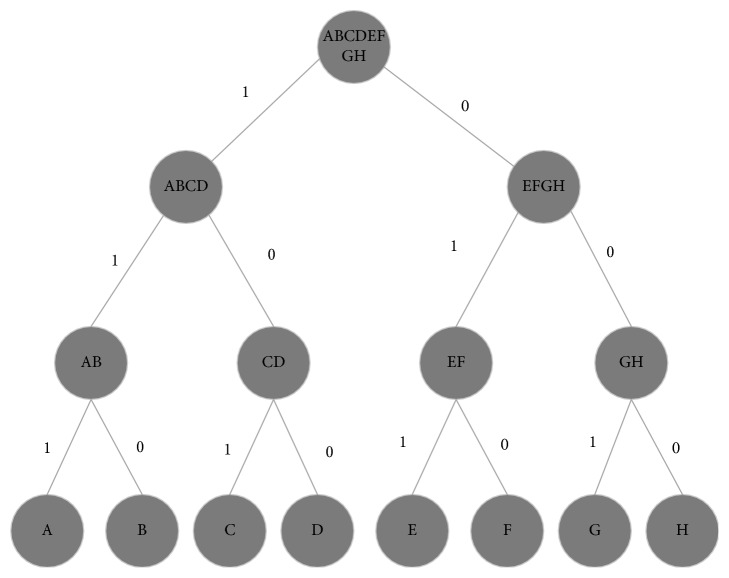
Prefix tree for symbol with the same appearance frequency.

Another issue in using Huffman coding occurs when all bytes or symbols have the same occurrence frequency, and the generated prefix tree has a log  2(*n*) depth, where *n* is the total number of symbols (*n*=256, for each file). The generated codeword for each symbol has a length in the amount of depth from the created prefix tree, log  2(256) = 8 bit. In this case, no compression was generated from the generated codeword.  Figure 2 illustrates the Huffman coding limitation for the same occurrence frequency, assuming that each character only needed 3 bits to be compiled (A = 000, B = 001, C = 010,…, H = 111).

Table 9 shows that the generated codeword has the same 3 bit length as the initial symbol. Therefore, no compression occurred during the process. If the value of occurrence frequency for each symbol is 5, then size of the original file (5 ∗ 8 ∗ 3 = 120 bit) will be the same as that of the compressed file (5 ∗ 8 ∗ 3 = 120 bit). This illustration is quite similar to the case of CT-MONO2-16-chest.dcm file, where the BFD values for the bytes have nearly the same value without centering on a much greater value. Consequently, the created prefix tree generates the same length of codeword as the initial bit length (8 bit), one fraction with 7-bit length and others with 9- and 10-bit lengths. Therefore, the size of the compressed file becomes larger than that of the original file.

### 4.2. Security Aspect

**Figure 3:**
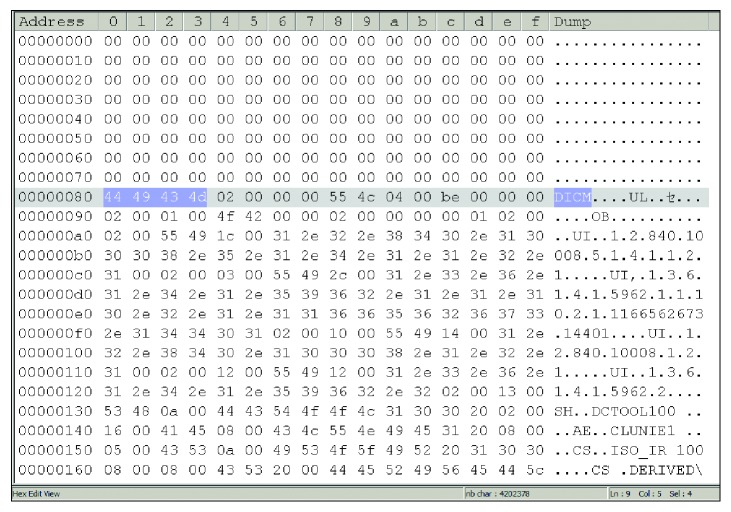
CT0011.dcm file hex content.

**Figure 4:**
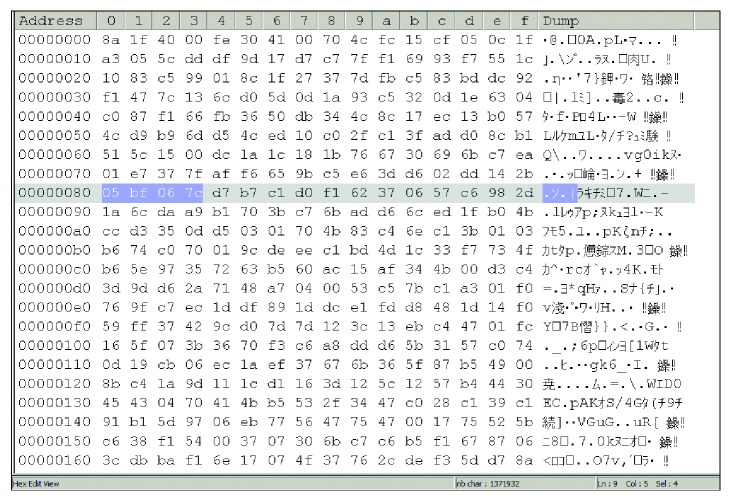
CT0011.HUF file hex content.

One of the problems in the security of the DICOM file is that the information of the DICOM file itself can be easily read by using general text editors, like Notepad or WordPad. This feature is a serious threat as even pixel data can be read by only with taking a few last bytes from the DICOM file. Figures 3 and 4 show the structure comparison between the original DICOM and the compressed files. The symbol character, 129 to 132 previously read as “DICM” on the DICOM file, is unreadable on the compressed file. The prefix “1.2.480…” previously was able to be read, which indicates that the DICOM file is no longer available in the compressed DICOM file.

All characters that can be directly read previously, such as patient's name, doctor's name, hospital, and date, change to unique characters in a compressed file. The set of last bytes no longer represents the pixel data from the original DICOM file. Now, interpreting the compressed DICOM file is difficult without decompressing the file, and the process for decompression is only known by the user. The worst thing that can be done by anyone on the compressed file is to change the structure or byte distribution from the file. This change may cause decompression that generates a file with a different bit structure from the original DICOM file. As a result, the generated file becomes unreadable in the DICOM viewer or corrupted. The result of the decompression process from the compressed file to be converted into the original DICOM file is shown in Table 10.

### 4.3. Huffman Coding Compression Time

**Figure 5:**
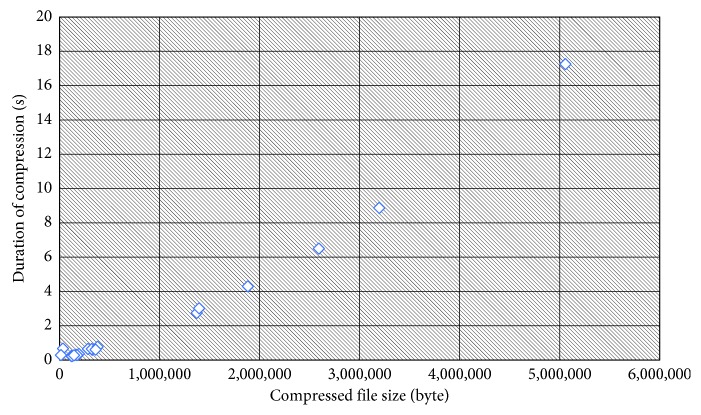
File size effect on duration of compression.

The duration of compression is proportional to the generated compressed file size. The larger the generated file size, the longer the time required for compression.  Figure 5 shows the effect of duration of compression on the generated file size.

In Huffman coding, inputted data are traversed when they receive a BFD value and when they are assigning a codeword for every symbol. If the input file size is defined as *n*, then the time needed will be 2 ∗ *n*. For the prefix tree, if the nodes and symbols are defined as |∑|, with the depth of log  2|∑|, then the prefix tree's size becomes 2*∗*|∑| (the total number of final nodes). From this state, we can obtain a traversed prefix tree for the collection of symbol codewords, which can be represented according to the construction time of the prefix tree's best and worst cases.

**Figure 6:**
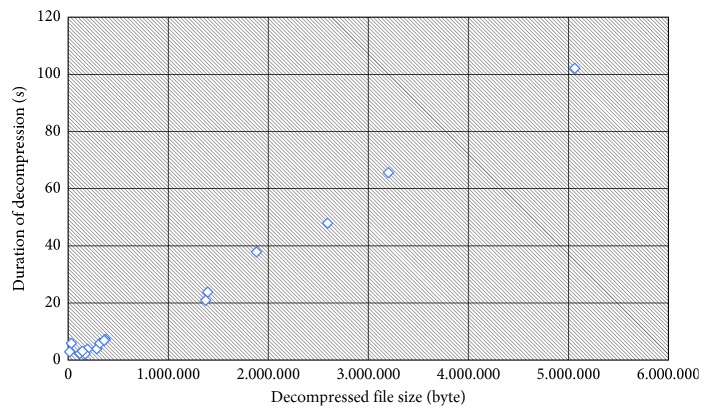
File size effect on duration of decompression.

The time required for decompressing depends on the compressed file size, which is caused by the search to decompress all the bits in the file from the initial to the last bit.  Figure 6 shows the effect of duration of decompression on the decompressed file size. Table 10 shows that a significantly longer time is required for the decompression process than for the compression time. For example, the CT0031.dcm file was decompressed for 101.93 seconds but compressed for only 17.25 seconds. However, the compression and decompression times are both proportional to the compressed size and not to the original file size.  Figure 6 displays the correlation graph between the compressed file sizes with the required decompression time.

### 4.4. Best Case Complexity

The best case in Huffman coding occurs when the constructed prefix tree forms a shape of perfectly height-balanced tree, where subtrees in the left and the right form one node that has similar height. In the perfectly height-balanced prefix tree, the traversed time for each symbol will take log  2|∑|. Therefore, every symbol will take |∑| *∗* log  2|∑|, and if we assume the length of the data is *n*, the best case complexity will become 2 *∗* *n*+|∑| *∗* log  2|∑| or *O*(*n*+|∑| *∗* log  2|∑|).

### 4.5. Worst Case Complexity

The worst case in Huffman coding occurs when the constructed prefix tree forms a shape of a degenerated or linear tree, which is called the unbalanced tree. This case takes time to reach one node as |∑|. Therefore, reaching a codeword for every symbol takes |∑|*∗*|∑|=|∑|^2^. Thus, the time complexity for worst case becomes 2 *∗* *n*+|∑|^2^ or *O*(*n*+|∑|^2^).

### 4.6. Comparison with JPEG2000

**Figure 7:**
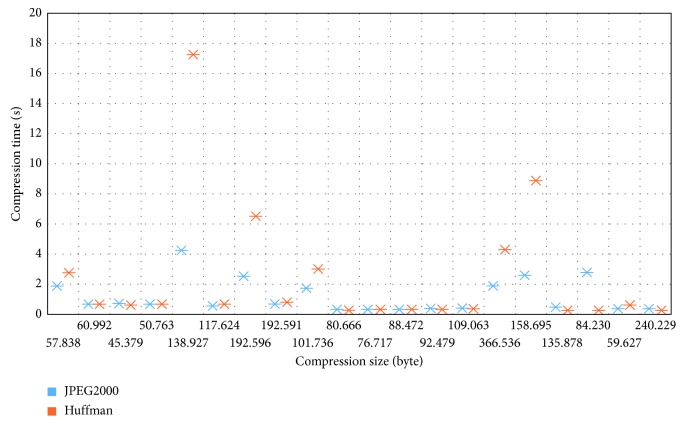
Performance comparison with JPEG2000.

Lossless JPEG2000 compression implementation was applied to the same set of DICOM files listed in Table 4 in order to get a benchmark performance comparison with Huffman coding.  Figure 7 depicts the results of comparing the compression time and size of JPEG2000 and Huffman coding. Overall, from the results, we can observe that Huffman coding compression performance was comparable with the standard JPEG2000 compression method with slightly faster compression time for CT images. However, JPEG2000 still outperforms Huffman coding on compressing large DICOM image such as CR, DR, and angiography.

Nevertheless, Huffman coding maintains the original file format and size while JPEG2000, being not backward compatible, changes the original size upon decompression. This comparable performance gives Huffman coding an advantage to be an alternative implementation of DICOM image compression in open PACS settings due to JPEG2000 proprietary implementation.

## 5. Conclusions

Huffman coding can generate compressed DICOM file with the value of the compression ratio up to 1 : 3.7010 and space savings of 72.98%. The compression ratio and percentage of the space savings are influenced by several factors, such as number of symbols or initial node used to create prefix tree and the pattern of BFD spread from the compressed file. The time required for compressing and decompressing is proportional to the compressed file size. That is, the larger the compressed file size, the longer the time required to compress or to decompress the file.

Huffman coding has time complexity, *O*(*n*+|∑|*∗*  log  2|∑|), and space complexity, *O*(|∑|), where *n* is the read input file size and |∑| is the number of symbols or initial nodes used to compile the prefix tree.

The structure of the compressed file cannot be easily interpreted as a DICOM file by using a general text editor, such as Notepad or WordPad. In addition, Huffman coding is able to compress and decompress with preserving any information in the original DICOM file.

There is a limitation which is at one time; the technique stops compressing when each symbol or node from the compressed file has the same occurrence frequency (all symbols have the same value of BFD).

Compression of the DICOM image can be conducted only in the pixel data from the image without changing the overall file structure, so the generated compressed file is still be able to be directly read as the DICOM file in the compressed pixel data size. Thus, for future research, encryption for the important information of DICOM files, such as patient ID, name, and date of birth, is considered to strengthen the secureness of the data.

Lastly, we also plan to further evaluate Huffman coding implementation for inclusion in the popular dcm4HCEE open PACS implementation. Such study will focus on transfer time, compression, and decompression until the image reading quality evaluation.

## Figures and Tables

**Table 1:** BFD for CT0011.dcm file.

Byte (hexadecimal)	Byte (decimal)	Frequency
00	0	2661671
01	1	613
02	2	724
03	3	49653
04	4	702819
…	…	…
FD	253	2
FE	254	49043
FF	255	887

**Table 2:** Codeword for byte distribution of CT0011.dcm file.

Byte (hexadecimal)	Byte (decimal)	Codeword
00	0	1
01	1	010000011110
02	2	010100111001
03	3	010111
04	4	00
…	…	…
FD	253	011111011100100011010
FE	254	010101
FF	255	011011001001

**Table 3:** Lookup table for CT0011.HUF file.

Symbol	Codeword
0	1
1	010000011110
2	010100111001
3	010111
4	00
…	…
253	011111011100100011010
254	010101
255	011011001001

**Table 4:** DICOM file specification used in the experiments.

File name	Number of frames	Size (byte)
CT0011.dcm	8	4.202.378
CT0012.dcm	2	1.052.902
CT0013.dcm	2	1.052.750
CT0014.dcm	2	1.053.770
CT0031.dcm	15	7.871.216
CT0032.dcm	1	527.992
CT0033.dcm	7	3.675.976
CT0034.dcm	1	528.012
CT0035.dcm	6	3.149.976
CT0051.dcm	1	208.402
CT0052.dcm	1	259.602
CT0055.dcm	1	208.416
CT0056.dcm	1	259.616
CT0059.dcm	1	362.378
CT0081.dcm	2	2.607.730
CT0110.dcm	9	4.725.954
CT-MONO2-8-abdo.dcm	1	262.940
CT-MONO2-16-ankle.dcm	1	525.436
CT-MONO2-16-brain.dcm	1	525.968
CT-MONO2-16-chest.dcm	1	145.136

**Table 5:** Compression results.

File name	Original size (byte)	No. of symbol/node	Compression time (s)	Compressed size (byte)
CT0011.dcm	4.202.378	254	2.75	1.371.932
CT0012.dcm	1.052.902	223	0.67	346.880
CT0013.dcm	1.052.750	209	0.60	328.241
CT0014.dcm	1.053.770	254	0.65	284.727
CT0031.dcm	7.871.216	256	17.25	5.057.232
CT0032.dcm	527.992	256	0.65	322.986
CT0033.dcm	3.675.976	256	6.50	2.592.421
CT0034.dcm	528.012	256	0.78	380.848
CT0035.dcm	3.149.976	256	3.02	1.395.825
CT0051.dcm	208.402	256	0.24	118.713
CT0052.dcm	259.602	256	0.29	145.620
CT0055.dcm	208.416	256	0.26	127.395
CT0056.dcm	259.616	256	0.31	164.952
CT0059.dcm	362.378	256	0.36	193.416
CT0081.dcm	2.607.730	256	4.30	1.882.801
CT0110.dcm	4.725.954	256	8.87	3.196.659
CT-MONO2-8-abdo.dcm	262.940	217	0.23	124.563
CT-MONO2-16-ankle.dcm	525.436	89	0.29	175.696
CT-MONO2-16-brain.dcm	525.968	256	0.61	360.802
CT-MONO2-16-chest.dcm	145.136	256	0.28	145.248

**Table 6:** Compression ratio value and space savings.

File name	Compression ratio	Space savings (%)
CT0011.dcm	3.0631	67.35
CT0012.dcm	3.0353	67.05
CT0013.dcm	3.2072	68.82
CT0014.dcm	3.7010	72.98
CT0031.dcm	1.5564	35.75
CT0032.dcm	1.6347	38.83
CT0033.dcm	1.4180	29.48
CT0034.dcm	1.3864	27.87
CT0035.dcm	2.2567	55.69
CT0051.dcm	1.7555	43.04
CT0052.dcm	1.7827	43.91
CT0055.dcm	1.6360	38.87
CT0056.dcm	1.5739	36.46
CT0059.dcm	1.8736	46.63
CT0081.dcm	1.3850	27.80
CT0110.dcm	1.4784	32.36
CT-MONO2-8-abdo.dcm	2.1109	52.63
CT-MONO2-16-ankle.dcm	2.9906	66.56
CT-MONO2-16-brain.dcm	1.4578	31.40
CT-MONO2-16-chest.dcm	0.9992	−0.08

**Table 7:** BFD for CT0013.dcm and CT0014.dcm file.

Byte (hexadecimal)	CT0013.dcm	CT0014.dcm
00	663477	698830
01	103	175
02	237	59
03	24806	14
…	…	…
11	5178	45
12	4703	18
13	4719	13
14	6152	6
…	…	…
FC	3	6
FD	2	5
FE	24489	91
FF	413	682

**Table 8:** BFD and codeword for CT-MONO2-16-chest.dcm file.

Byte (hexadecimal)	BFD	Codeword
00	938	0010110
01	495	01001000
02	532	01100111
03	495	01001001
…	…	…
7D	381	111111110
7E	472	00110011
7F	398	00000011
80	222	001001110
…	…	…
FC	266	100000000
FD	307	101001010
FE	210	1011100011
FF	117	1011100010

**Table 9:** Codeword for symbol with the same occurrence frequency.

Symbol	Codeword
A	111
B	110
C	101
D	100
E	011
F	010
G	001
H	000

**Table 10:** Decompression results.

File name	Original size (byte)	Time (s)	Decompressed size (byte)
CT0011.dcm	1.371.932	21.04	4.202.378
CT0012.dcm	346.880	5.92	1.052.902
CT0013.dcm	328.241	5.66	1.052.750
CT0014.dcm	284.727	4.08	1.053.770
CT0031.dcm	5.057.232	101.93	7.871.216
CT0032.dcm	322.986	6.06	527.992
CT0033.dcm	2.592.421	48.05	3.675.976
CT0034.dcm	380.848	7.18	528.012
CT0035.dcm	1.395.825	23.91	3.149.976
CT0051.dcm	118.713	2.41	208.402
CT0052.dcm	145.620	2.93	259.602
CT0055.dcm	127.395	2.62	208.416
CT0056.dcm	164.952	3.38	259.616
CT0059.dcm	193.416	3.83	362.378
CT0081.dcm	1.882.801	37.82	2.607.730
CT0110.dcm	3.196.659	65.76	4.725.954
CT-MONO2-8-abdo.dcm	124.563	2.28	262.940
CT-MONO2-16-ankle.dcm	175.696	2.34	525.436
CT-MONO2-16-brain.dcm	360.802	7.04	525.968
CT-MONO2-16-chest.dcm	145.248	3.01	145.136

## Data Availability

The partial data are obtained from publicly available medical image data from http://mmnt.net, while a small set of private data are obtained from local hospital after anonymizing the patient information details.
